# The burden of nonalcoholic steatohepatitis (NASH) in the United States

**DOI:** 10.1186/s12876-023-02726-2

**Published:** 2023-04-05

**Authors:** Elliot B. Tapper, Nancy Krieger, Raymond Przybysz, Nate Way, Jennifer Cai, Dion Zappe, Sarah Jane McKenna, Garth Wall, Nico Janssens, Maria-Magdalena Balp

**Affiliations:** 1 University of Michigan, Ann Arbor, MI USA; 2 Novartis Pharmaceuticals Corp, East Hanover, NJ USA; 3 Cerner Enviza, Malvern, PA USA; 4 Novartis Business Services Centre, Dublin, Ireland; 5 Novartis Pharma AG, Basel, Switzerland

**Keywords:** Nonalcoholic fatty liver disease, NAFLD, Type 2 diabetes, Burden of illness, US

## Abstract

**Background:**

There is limited data on the comparative economic and humanistic burden of non-alcoholic steatohepatitis (NASH) in the United States. The objective was to examine the burden of disease comparing NASH to a representative sample of the general population and separately to a type 2 diabetes mellitus (T2DM) cohort by assessing health-related quality of life (HRQoL) measures, healthcare resource use (HRU) and work productivity and activity impairment (WPAI).

**Methods:**

Data came from the 2016 National Health and Wellness Survey, a nationally representative patient-reported outcomes survey conducted in the United States. Respondents with physician-diagnosed NASH, physician-diagnosed T2DM, and respondents from the general population were compared. Humanistic burden was examined with mental (MCS) and physical (PCS) component summary scores from the Short-Form (SF)-36v2, concomitant diagnosis of anxiety, depression, and sleep difficulties. Economic burden was analysed based on healthcare professional (HCP) and emergency room (ER) visits, hospitalizations in the past six months; absenteeism, presenteeism, overall work impairment, and activity impairment scores on WPAI questionnaire. Bivariate and multivariable analysis were conducted for each outcome and matched comparative group.

**Results:**

After adjusting for baseline demographics and characteristics, NASH (*N* = 136) compared to the matched general population cohort (*N* = 544), reported significantly lower (worse) mental (MCS 43.19 vs. 46.22, *p* = 0.010) and physical (PCS 42.04 vs. 47.10, *p* < 0.001) status, higher % with anxiety (37.5% vs 25.5%, *p* = 0.006) and depression (43.4% vs 30.1%, *p* = 0.004), more HCP visits (8.43 vs. 5.17), ER visits (0.73 vs. 0.38), and hospitalizations (0.43 vs. 0.2) all *p*’s < 0.05, and higher WPAI scores (e.g. overall work impairment 39.64% vs. 26.19%, *p* = 0.011). NASH cohort did not differ from matched T2DM cohort (*N* = 272) on mental or work-related WPAI scores, but had significantly worse physical status (PCS 40.52 vs. 44.58, *p* = 0.001), higher % with anxiety (39.9% vs 27.8%, *p* = 0.043), more HCP visits (8.63 vs. 5.68, *p* = 0.003) and greater activity impairment (47.14% vs. 36.07%, *p* = 0.010).

**Conclusion:**

This real-world study suggests that burden of disease is higher for all outcomes assessed among NASH compared to matched general controls. When comparing to T2DM, NASH cohort has comparable mental and work-related impairment but worse physical status, daily activities impairment and more HRU.

**Supplementary Information:**

The online version contains supplementary material available at 10.1186/s12876-023-02726-2.

## Introduction

Nonalcoholic steatohepatitis (NASH), the severe form of nonalcoholic fatty liver disease (NAFLD), can become life-threatening, leading to the development of a range of complications including cirrhosis and hepatocellular carcinoma [1]. Patients with NASH commonly experience little or no symptoms in the early stages of the condition, which can explain lower levels of diagnosis. As disease progresses various non-specific symptoms are reported and are attributable to comorbidities rather than NASH itself [2].

As a result of the possible asymptomatic presentation, low disease awareness, low rate of liver biopsy (the gold standard for NASH diagnosis) and no approved specific therapies, the prevalence and burden of NASH may be underestimated [3–6]. Some published studies describe a substantial healthcare resource use (HRU) [7], impairments in daily activities [8], and reduced health-related quality of life (HRQoL) among NASH patients [9]. However, studies examining the burden of NASH in the United States (US) are limited and more studies are needed to comprehensively examine the humanistic and economic burden of NASH compared to other conditions [10].

We aim to address this gap using data from the 2016 National Health and Wellness Survey (NHWS), a nationally representative patient-reported outcomes survey conducted in the US. The overall objective of this study was to assess the humanistic (HRQoL measures) and the economic burden (impact on work and HRU) of NASH in comparison to general population and type 2 diabetes mellitus (T2DM) respectively. In addition, the study assessed the prevalence of diagnosed NASH in the US. This study complements the identical burden of disease study conducted within the NHWS from five European countries (EU5) by the same authors [10].

## Methods

### Data source

Data came from the 2016 National Health and Wellness Survey (NHWS), a multinational internet-based patient-reported outcomes survey including 97,503 respondents across the US (collected during March-August 2016). Stratified random sampling was used to ensure that the sample is representative of the general US adult population across gender, age and race/ethnicity. The majority of NHWS participants completed the survey through opt-in online platforms (e.g., MySurvey.com), the remainder of participants (less than 10%) were recruited offline. The Pearl Institutional Review Board assessed the NHWS and awarded it exemption. Informed consent was obtained from all participants [11].

### Study cohorts

Three cohorts were created for this study by extracting the following cases from the NHWS: respondents with physician-diagnosed NASH; general population respondents (a representative sample of the general population with various health status); and respondents with physician-diagnosed T2DM. All respondents answered questions related to socio-demographics, clinical characteristics, patient-reported outcomes and HRU (see Supplementary Materials). All analyses, except those used to estimate the diagnosed prevalence of NASH, excluded respondents with a physician diagnosis of hepatitis B, hepatitis C, or cirrhosis.

The analysis included two versions of the Charlson Comorbidity Index (CCI): standard CCI [12] and adjusted CCI. Diabetes, diabetes with end organ damage, mild liver disease, peripheral vascular disease, myocardial infarction (heart attack), and congestive heart failure were excluded from the adjusted CCI (for explanation, see Statistical Analysis).

### Patient-reported outcomes

#### Health-Related Quality of Life (HRQoL)

Respondents completed the Medical Outcomes Study 36-Item Short Form Survey Instrument Version 2 (SF-36v2) a generic measure of health status which allows comparisons among various diseases. Physical Component Summary (PCS) score and Mental Component Summary (MCS) score were calculated using a norm-based algorithm derived from an US general population survey. The PCS and MCS scores range from 0–100. Lower scores indicate worse status. The minimal important difference (MID) for PCS and MCS scores is 3.0 [13–15].

SF-6D utility score (MID = 0.041) was derived from the PCS and MCS scores. Respondents also completed the EQ-5D-5L [16] questionnaire which allows the calculation of the EQ-5D utility score (MID = 0.074). Both utility scores range from 0 (death) to 1 (perfect health) [15–18].

#### Psychological comorbidities

Percentage of respondents reporting diagnosed anxiety, depression, and sleep difficulties, respectively, in the past 12 months was analyzed in each study cohort.

#### Healthcare Resource Use (HRU)

All respondents reported the number of healthcare visits over the previous six months divided into four categories: healthcare professional (HCP) visits (e.g., general practitioner/family practitioner, internist, allergist, cardiologist, etc.), non-traditional provider visits (e.g., acupuncturist, herbalist, nutritionist, massage therapist, etc.), emergency room (ER) visits, and hospitalizations. The supplementary Materials contain the list of all visit types.

HCP visits were evaluated by the total amount of visits to any HCP and the number of visits to specialist HCPs. Non-traditional provider visits were analyzed by the number of different non-traditional provider types visited and the number of such visits (≥ 1 vs. none).

#### Work productivity and activity impairment

Work and activity impairment in the previous 7 days were assessed on the Work Productivity and Activity Impairment Questionnaire-General Health (WPAI-GH). Four scores (absenteeism, presenteeism, overall work impairment and activity impairment) were calculated and reported on a scale from 0 to 100%, where higher scores denote higher impairment [19]. Only employed respondents completed the questions related to work.

### Statistical analysis

The socio-demographics and clinical characteristics of the three unmatched cohorts were analyzed with descriptive statistics. Diagnosed prevalence of NASH in the US adult population was calculated using sampling weights based on age, gender, race/ethnicity, and education from an official 2016 US national census source [20].

The NASH cohort was compared to a matched general population cohort and separately to a matched T2DM cohort using matched bivariate analyses. A propensity score matching approach based on age, gender, education, income, smoking behavior, current alcohol use, current exercise behavior, and adjusted CCI was used to construct the general population and T2DM cohorts (see Supplementary Materials).

Chi-square tests were performed for bivariate comparisons for categorical variables and one-way ANOVAs testing for continuous variables. Statistical significance was defined as a two-sided p-value of less than 0.05.

Propensity score matching was used to control for confounding variables. After the propensity score matching was done, matched group comparisons (NASH vs. matched general population and NASH vs. matched T2DM separately) were performed by modeling each outcome individually, using multivariable analysis. No matching criteria remained unbalanced in NASH versus general population comparisons nor in NASH versus T2DM comparisons, therefore no covariates were used in NASH versus general population multivariable comparisons.

As a proportion of NASH patients had comorbid T2DM and to control for T2DM severity, several T2DM-related variables (current prescription for T2DM, presence of at least one relevant diagnosed heart or blood condition and at least one diagnosed T2DM related comorbidity) were used as covariates in the NASH versus T2DM multivariable regression models. (Supplementary Materials).

Various regression models (e.g. linear, binary logistic, generalized linear models with negative binomial distribution or log-link models) were used for the different outcomes of interest. All results are reported as adjusted means. Statistical significance was defined as a two-sided p-value of less than 0.05.

## Results

### NASH cohort description and prevalence

The initial unmatched cohorts described in Table 1 comprised 136 patients with NASH, 96,108 respondents from the general population and 8,175 patients with T2DM. The mean (SD) age of NASH respondents at the time of the survey was 49.9 (13.4) years, 61.8% female and 78.0% non-Hispanic white. Among NASH, 66.9% had high BMI suggesting obesity, 50.0% hypertension, 33.1% had T2DM and 22.1% a CCI above 3. The general population and T2DM unmatched cohorts had a mean (SD) age of 46.3 (17.2) and 60.1 (12.5) years respectively with 57.6% and 46.7% female. A high BMI suggestive of obesity was reported among 29.7% of the general population cohort and 60.4% of the T2DM cohort.

**Table 1:** Socio-demographics and clinical characteristics of the three unmatched cohorts: NASH, general population, T2DM

		**Unmatched Cohorts**		
		**NASH** ***N* = 136**	**General Population** ***N* = 96,108**	**T2DM** ***N* = 8,175**
		**N (%)**	**N (%)**	**N (%)**
**Demographics**				
Gender	*Female*	84 (61.8%)	55,361 (57.6%)	3,821 (46.7%)
	*Male*	52 (38.2%)	40,747 (42.4%)	4,354 (53.3%)
Race/ethnicity	*Non-Hispanic White*	106 (78.0%)	61,367 (63.8%)	5,948 (72.8%)
	*Non-Hispanic Black*	1 (0.7%)	9,139 (9.5%)	764 (9.3%)
	*Hispanic*	13 (9.6%)	9,999 (10.4%)	595 (7.3%)
	*Other*	16 (11.8%)	15,603 (16.2%)	868 (10.6%)
Age, Mean (SD), years		49.9 (13.4)	46.3 (17.2)	60.1 (12.5)
Education	*Less than university degree*	74 (54.4%)	51,870 (54.0%)	4,910 (60.1%)
	*University degree or higher*	62 (45.6%)	44,072 (45.9%)	3,262 (39.9%)
	*Decline to answer*	0 (0.0%)	166 (0.2%)	3 (0.0%)
Income	*Below region median income*	60 (44.1%)	39,854 (41.5%)	3,938 (48.2%)
	*At or above region median income*	73 (53.7%)	50,353 (52.4%)	3,855 (47.2%)
	*Decline to answer*	3 (2.2%)	5,901 (6.1%)	382 (4.7%)
Health insurance status	*Insured*	120 (88.2%)	86,933 (90.4%)	7,771 (95.1%)
	*Not insured*	16 (11.8%)	9,175 (9.5%)	404 (4.9%)
Health insurance type	*Employer-sponsored*	58 (42.6%)	46,146 (48.0%)	2,592 (31.7%)
	*Other*	62 (45.6%)	38,448 (40.0%)	5,126 (62.7%)
	*Not sure/decline to answer*	16 (11.8%)	11,514 (12.0%)	457 (5.6%)
Currently employed		71 (52.2%)	55,585 (57.8%)	3,030 (37.1%)
**Patient Characteristics**				
BMI	*Obese*	91 (66.9%)	28,523 (29.7%)	4,937 (60.4%)
	*Overweight*	22 (16.2%)	29,155 (30.3%)	2,215 (27.1%)
	*Normal weight*	16 (11.8%)	32,331 (33.6%)	783 (9.6%)
	*Underweight*	2 (1.5%)	2,546 (2.6%)	39 (0.5%)
	*Unknown*	5 (3.7%)	3,553 (3.7%)	201 (2.5%)
Smoking behavior	*Current smoker*	20 (14.7%)	13,615 (14.2%)	1,185 (14.5%)
	*Former smoker*	38 (27.9%)	23,636 (24.6%)	2,961 (36.2%)
	*Never smoker*	78 (57.3%)	58,857 (61.2%)	4,029 (49.3%)
Current alcohol use	*None*	59 (43.4%)	32,766 (34.1%)	3,681 (45.0%)
	*Yes, less than daily*	70 (51.5%)	58,411 (60.8%)	4,184 (51.2%)
	*Yes, daily*	7 (5.1%)	4,931 (5.1%)	310 (3.8%)
Current exercise behavior	*No exercise: 0 days in past month*	57 (41.9%)	33,019 (34.4%)	4,211 (51.5%)
	*Low exercise: 1–5 days in past month*	34 (25.0%)	19,951 (20.8%)	1,249 (15.3%)
	*Moderate exercise: 6–11 days in past month*	18 (13.2%)	12,896 (13.4%)	812 (9.9%)
	*High exercise: 12* + *days in past month*	27 (19.8%)	30,242 (31.5%)	1,903 (23.3%)
**Comorbidities**				
CCI^a^	*CCI: 0*	48 (35.3%)	74,352 (77.4%)	0 (0.00%)
	*CCI: 1*	40 (29.4%)	12,824 (13.3%)	5,044 (61.7%)
	*CCI: 2*	18 (13.2%)	5,271 (5.5%)	1,511 (18.5%)
	*CCI: 3* +	30 (22.1%)	3,661 (3.8%)	1,620 (19.8%)
Adjusted CCI^a^	*CCI: 0*	73 (53.7%)	80,899 (84.2%)	5,356 (65.5%)
	*CCI: 1*	27 (19.9%)	8,519 (8.9%)	1,451 (17.7%)
	*CCI: 2*	15 (11.0%)	4,386 (4.6%)	814 (10.0%)
	*CCI: 3* +	21 (15.4%)	2,304 (2.3%)	554 (6.8%)
Physician diagnosis of:				
Hypertension		68 (50.0%)	23,902 (24.9%)	5,560 (68.0%)
One or more heart or blood conditions^b^		92 (67.6%)	33,531 (34.9%)	6,656 (81.4%)
Physician diagnosis of T2DM		45 (33.1%)	8,175 (8.5%)	8,175 (100.0%)
One or more T2DM-related complication^c^		20 (14.7%)	2,392 (2.5%)	2,392 (29.3%)
Treatment for T2DM:				
All prescription treatments		35 (25.7%)	6,889 (7.2%)	6,889 (84.3%)
Insulin prescription		14 (10.3%)	2,034 (2.1%)	2,034 (24.9%)
Non-insulin prescription		31 (22.8%)	6,218 (6.5%)	6,218 (76.1%)

*Abbreviations*: *NASH* nonalcoholic steatohepatitis, *T2DM* type 2 diabetes mellitus, *SD* standard deviation, *BMI* body mass index, *CCI* Charlson Comorbidity Index

^a^CCI—higher scores indicate greater comorbid burden on patient

^b^Included physician diagnosis of one or more of the following: atherosclerosis (coronary artery disease), congestive heart failure, heart attack, stroke, mini-stroke/transient ischemia attack, hypertension (high blood pressure), high cholesterol, peripheral arterial disease, peripheral vascular disease, angina

^c^Included physician diagnosis of one or more of the following: macular edema or diabetic retinopathy, kidney disease, foot or leg ulcer, neuropathic pain, end organ damage due to diabetes

The 2016 prevalence of diagnosed NASH among the US adult population was 0.15%.

### Burden of NASH vs. Matched General Population

The bivariate analysis of NASH versus the matched general population (*N* = 544) showed worse HRQoL, higher HRU and higher (worse) WPAI scores for NASH (Supplementary Table 1). The results of the multivariable analysis presented below confirm the bivariate results.

**Fig. 1:**
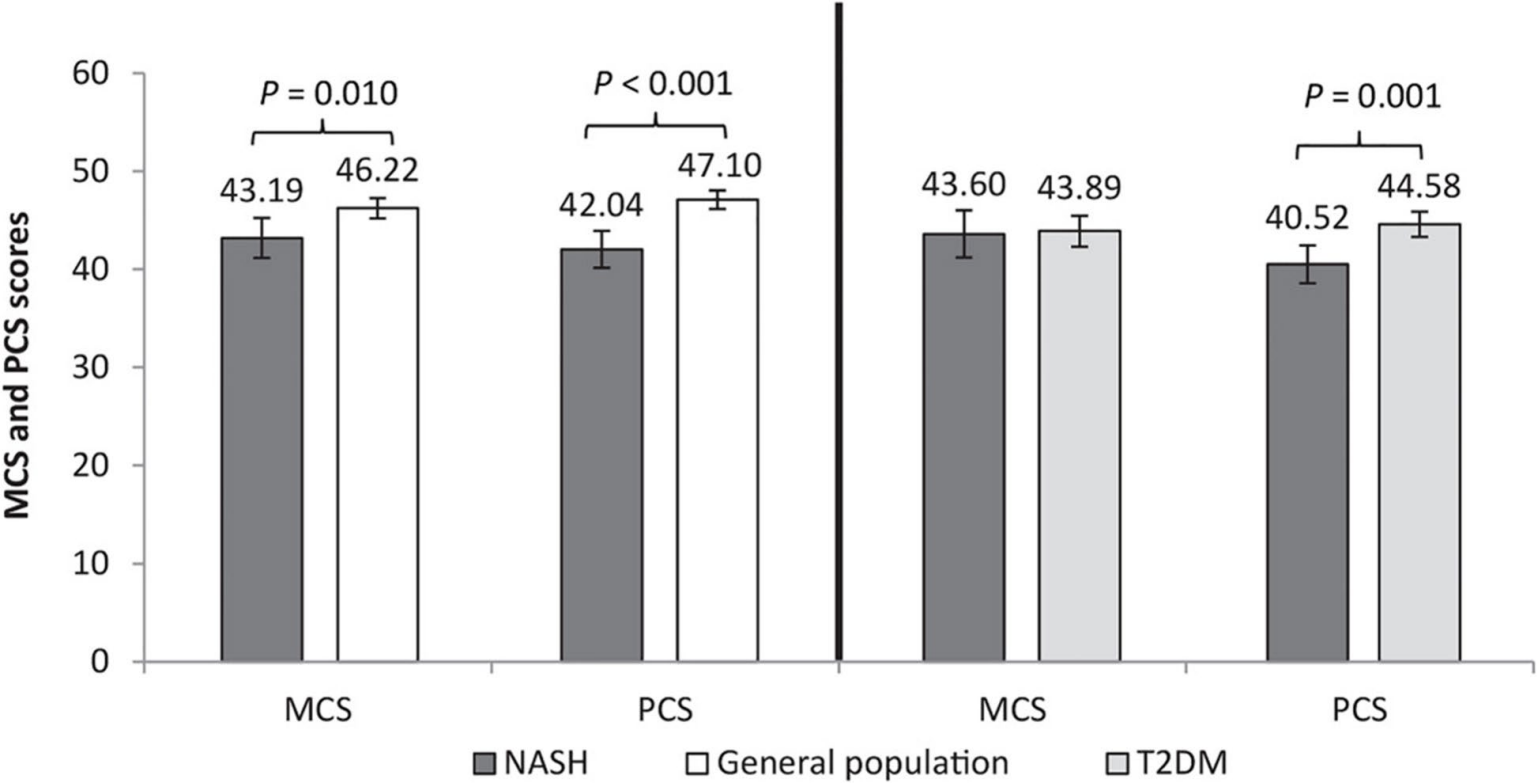
MCS and PCS scores: NASH vs. matched general population and NASH vs. matched T2DM. Note: The above scores are multivariable results and displayed as adjusted means. Standard error and upper and lower 95% confidence intervals are presented in Supplementary Table 2; *p*-values represent significance of the regression coefficient in the regression model for each comparison (NASH vs matched general population and NASH vs matched T2DM). Lower scores mean lower status. Abbreviations: NASH, non-alcoholic steatohepatitis; MCS, mental component summary; PCS, physical component summary; T2DM, type 2 diabetes mellitus

Compared to the matched general population, NASH patients had significantly lower (worse) mental and physical component summary scores (MCS 43.19 vs. 46.22, *p* = 0.010; PCS 42.04 vs. 47.10, *p* < 0.001) (Fig. 1). SF-6D and EQ-5D scores were also significantly lower, (0.63 vs. 0.69, *p* < 0.001 and 0.72 vs. 0.78, *p* < 0.001), respectively (Supplementary Fig. 1 and Supplementary Table 2). Apart from EQ-5D utility score, differences on all scores exceeded accepted MIDs.

**Fig. 2:**
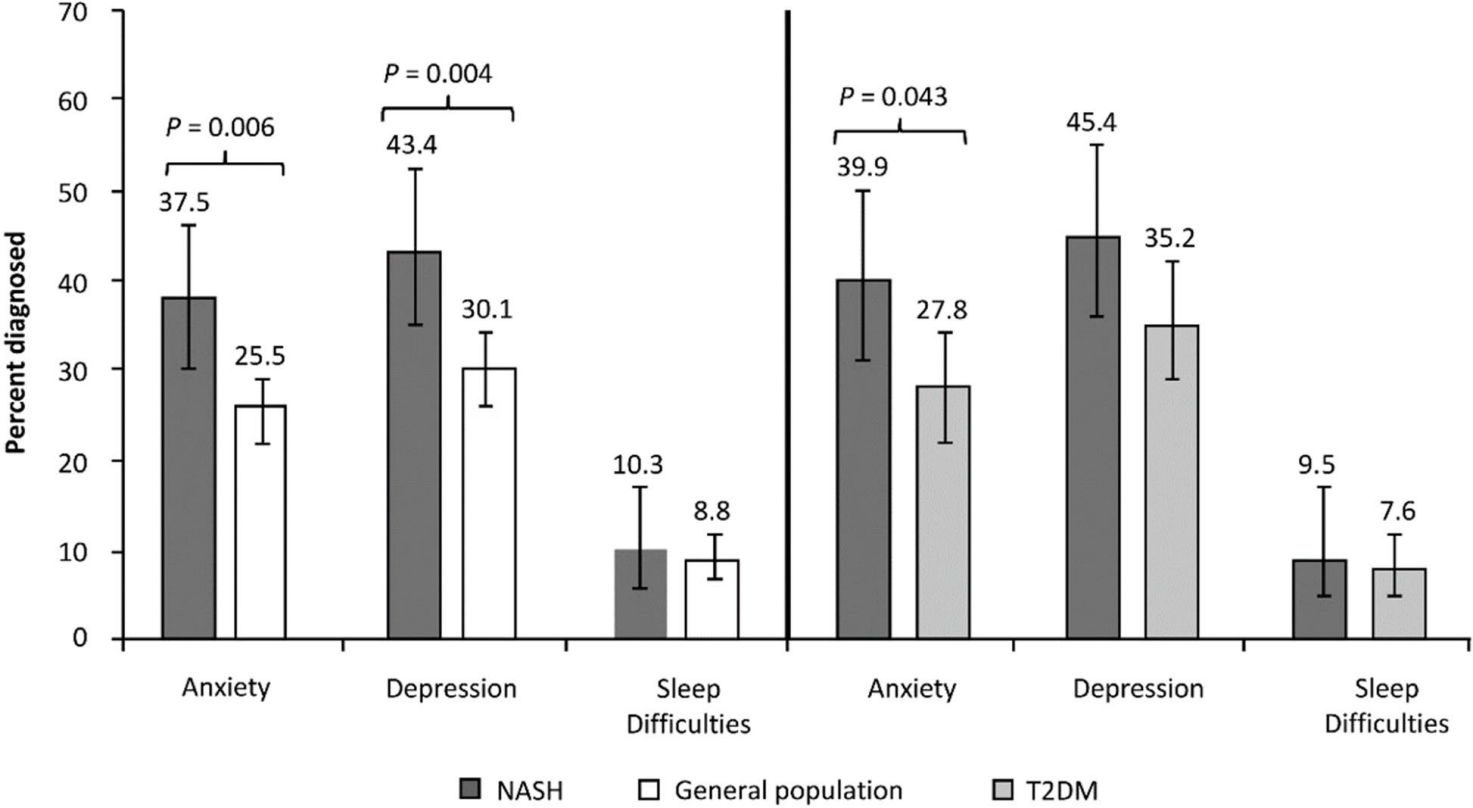
Patients with psychological comorbidities: NASH vs. matched general population and NASH vs. matched T2DM. Note: The above scores are multivariable results reported as percentage of patients with diagnosed anxiety, depression, or sleep difficulties; *p*-values represent significance of the regression coefficient in the regression model for each matched comparison. Standard error and upper and lower 95% confidence intervals are presented in Supplementary Table 3. Abbreviations: NASH, nonalcoholic steatohepatitis; T2DM, type 2 diabetes mellitus

More NASH patients reported anxiety (37.5% vs. 25.5%, *p* = 0.006) and depression (43.4% vs. 30.1%, *p* = 0.004), but sleep difficulties did not differ between cohorts (Fig. 2).

**Fig. 3:**
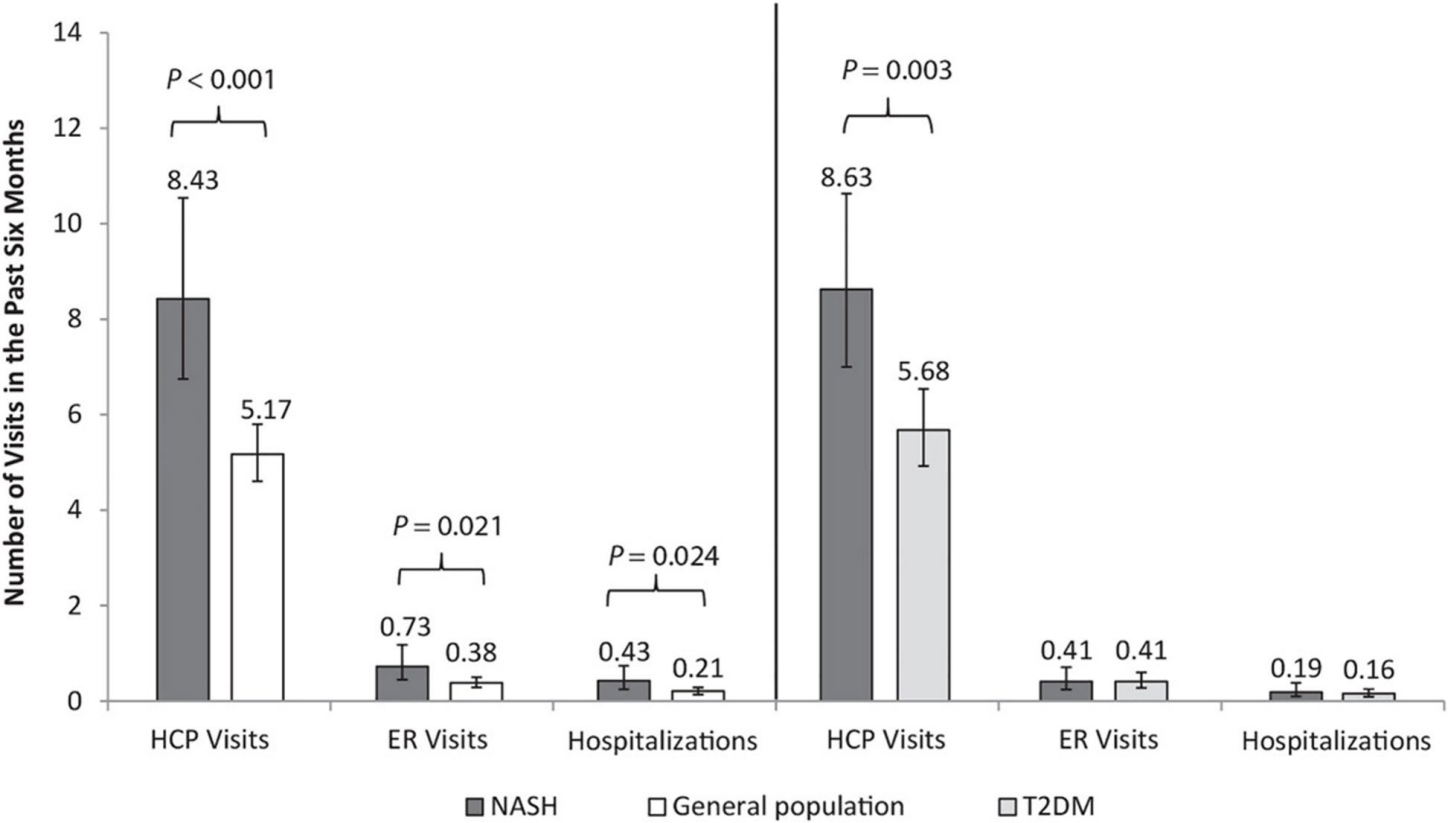
HRU: NASH vs. matched general population and NASH vs. matched T2DM. Note: Multivariable results displayed as adjusted mean over the past 6 months; *p*-values represent significance of the regression coefficient in the regression model for each matched comparison. Standard error and upper and lower 95% confidence intervals are presented in Supplementary Table 6. Abbreviations: ER, emergency room; HCP, healthcare professional; HRU, healthcare resource use; NASH, nonalcoholic steatohepatitis; T2DM, type 2 diabetes mellitus

In terms of HRU, there were more visits to an HCP (8.43 vs. 5.17, *p* < 0.001), ER visits (0.73 vs. 0.38, *p* = 0.021), and hospitalizations (0.43 vs. 0.21, *p* = 0.024) for NASH compared to the matched general population cohort (Fig. 3) and NASH patients reported more specialty visits (*p’s* < 0.05), for example, more general/family medicine provider visits (1.49 vs. 1.14, *p* = 0.040), hepatologist visits (0.10 vs. 0.02, *p* = 0.022), and gastroenterologist visits (0.38 vs. 0.12 *p* < 0.001) (Supplementary Table 5).

Furthermore, NASH patients reported visiting more types of non-traditional providers (1.21 vs. 0.66, *p* < 0.001) and a greater percentage of them reported at least one such visit (66.2% vs. 45.0%, *p* < 0.001) (Supplementary Table 6).

**Fig. 4:**
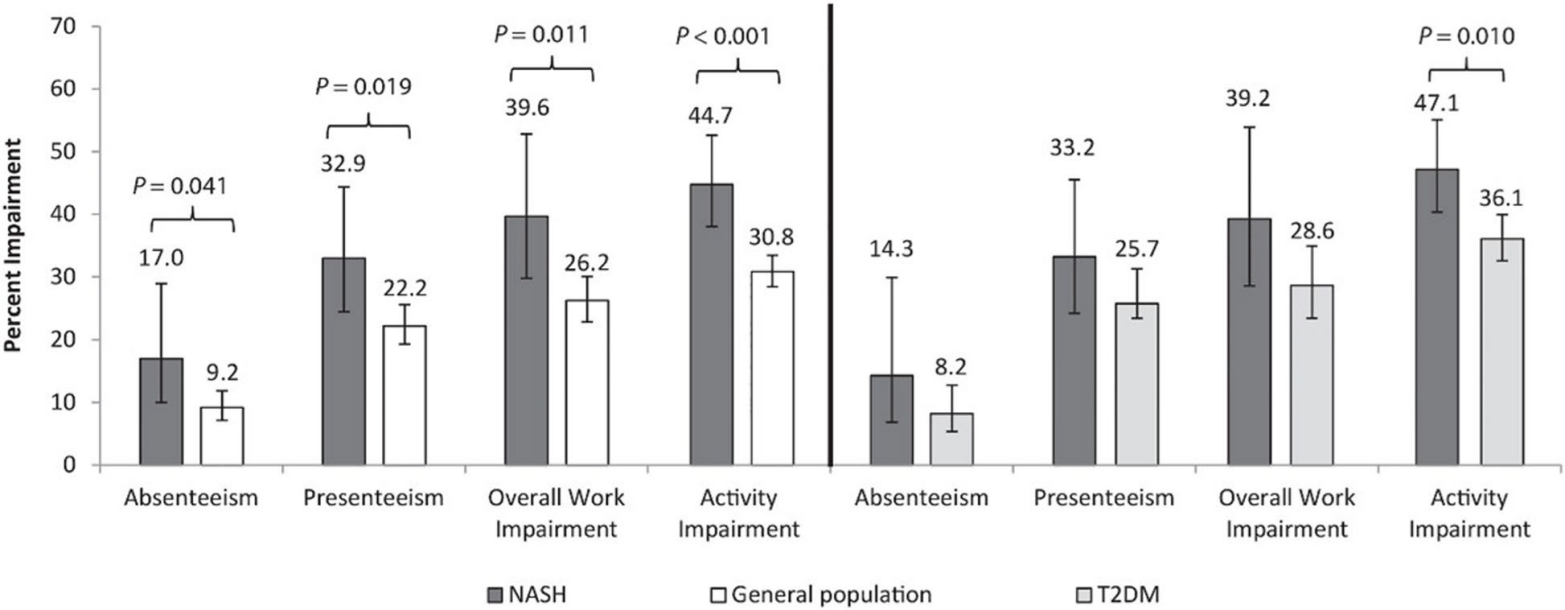
WPAI scores: NASH vs. matched general population and NASH vs. matched T2DM. Note: Multivariable results presented as adjusted % means**;** *p*-values represent significance of the regression coefficient in the regression model for each matched comparison. Absenteeism, presenteeism, and overall work impairment reported only by employed respondents. Standard error and upper and lower 95% confidence intervals are presented in Supplementary Table 4. Higher scores mean higher impairment. Abbreviations: NASH, nonalcoholic steatohepatitis; T2DM, type 2 diabetes mellitus; WPAI, Work Productivity and Activity Impairment

Impairment of work and activity on WPAI was significantly higher for NASH patients compared to matched general population cohort, including absenteeism (17.0% vs. 9.2%, *p* = 0.041) and presenteeism (32.9% vs. 22.2%, *p* = 0.019), as well as overall work impairment (39.6% vs. 26.2%, *p* = 0.011), and activity impairment (44.7% vs. 30.8%, *p* < 0.001) (Fig. 4).

### Burden of NASH vs. Matched T2DM Population

In the bivariate analysis, the NASH cohort was compared to a matched T2DM cohort of *N* = 272. The NASH cohort reported more HCP visits, non-traditional provider visits and higher absenteeism, (*p’s* < 0.05) than T2DM cohort but had similar scores on the other measures assessed (Supplementary Table 7). Multivariable results are presented below and present certain differences from the bivariate results.

NASH patients had a significantly lower (worse) PCS score than T2DM patients (40.52 vs. 44.58, *p* = 0.001; Fig. 1) and the difference exceeded MID. Mental status (MCS), SF-6D, and EQ-5D utility scores did not differ between the two groups (Fig. 1, Supplementary Fig. 1 and Supplementary Table 2).

A significantly higher percentage of NASH patients vs. T2DM reported anxiety (39.9% vs. 27.8%, *p* = 0.043). The difference on depression and sleep difficulties was not significant. (Fig. 2).

In terms of HRU, NASH patients reported more HCP visits (8.63 vs. 5.68, *p* = 0.003; (Fig. 3) and specialist visits (6.97 vs. 3.98, *p* = 0.001) than T2DM patients; Supplementary Table 8). More NASH patients reported at least one non-traditional provider visit (63.2% vs. 48.3%, *p* = 0.018) and visited more types of such providers (1.11 vs. 0.72, *p* = 0.005) compared to T2DM patients. There was no significant difference on any other HRU outcomes (Fig. 4; Supplementary Table 8).

The NASH cohort had higher absenteeism, presenteeism and overall work impairment scores than T2DM but the difference was not significant, while the activity impairment score was significantly higher for NASH (47.1% vs. 36.1%, *p* = 0.010; Fig. 4).

## Discussion

The results of this study suggest that NASH is associated with a significant humanistic and economic burden, in comparison to matched samples from the general population and T2DM respectively. These novel data come from the first study assessing the comparative burden of NASH in a nationally representative sample of the US population which complements the similar one conducted in NHWS EU5 [10]. The comparative burden is of interest as it allows first to position NASH versus a matched cohort representing the general population (a mix of individuals with various health status at a given moment) and secondly to compare specifically to T2DM, a condition with a substantial, well-characterized burden [21–24]. These results bring new evidence about the burden and comparative burden of NASH from representative patient-reported cohorts and in addition allow comparison with cohorts outside the US.

To our knowledge this is the first study to report prevalence of diagnosed NASH (0.15%) using a representative sample of the adult US population and appropriate statistical methods. This may offer a more accurate estimate than previously published studies which provide highly variable results, because of variations in cohort definition, sample size or methodological issues (e.g. no adequate extrapolation to the general population) [3–6]. A higher prevalence rate (0.29%) was reported in the NHWS EU5 study [10] that used the same methodology as the current study.

The results of the matched comparison to the general population cohort suggest that NASH is associated with a significantly higher humanistic and economic burden. After adjusting for covariates, the NASH cohort reported significantly worse HRQoL, more patients with diagnosed anxiety and depression, more healthcare resource use, higher work and activity impairment than the general population.

The multivariable analysis of NASH versus the matched T2DM cohort show that NASH patients reported significantly and clinically meaningful worse physical status, higher activity impairment, more HCP and non-traditional provider visits. More NASH patients reported diagnosed anxiety. The other study outcomes were not different for NASH and T2DM cohorts suggesting a similar burden.

These findings present similarities but also some differences to the identical study conducted in NHWS EU5 [10]. First, the profile of the NASH cohorts presents some differences between US and EU5 [10]: younger in US (mean age 49.9 vs 54.5), more female (61.8% vs 42.9%), higher % of patients with BMI suggesting obesity (66.9% vs 46.7%), and more with concomitant T2DM (33.1% vs 22.8%).

In both geographies NASH patients compared to the matched general population cohort reported worse mental and physical status, more anxiety and depression, higher WPAI scores and higher HRU. Compared to the matched general population significantly more NASH patients in EU5 reported sleep difficulties, while this outcome was similar among the compared cohorts in US.

In both geographies, NASH compared to the T2DM cohort reported significantly more HCP visits and similar results for depression, sleep difficulties and work-related scores. There were differences for the other outcomes in US and EU5. In US, NASH patients reported significantly worse physical status, more anxiety and activity impairment than T2DM, while in EU5 they had worse mental status, more ER visits, and hospitalizations.

While many of these findings show similar burden of NASH, independent of geographies, we assume that some of the differences in results could be generated by the differences in healthcare systems.

The results of our study complement results reported in previous research conducted in the US. According to our knowledge when the study was finalized, no previous work has analyzed the comparative burden of NASH in a nationally representative US sample. Many of the previous studies might have limitations due design or definition of population based on liver biopsy and histology [8, 25, 26]. Also, as some studies do not differentiate NASH from NAFLD and many studies included patients with cirrhosis whose outcomes are particularly poor, comparisons between results from prior studies and the current study may be difficult to interpret [7, 27]. Furthermore, no other published study compared NASH patients to matched controls (T2DM patients or general population) in the US, therefore this study provides for the first time such valuable insights. Alternatively, other studies assessed the burden of NASH in the context of other chronic liver diseases [7, 27, 28], or comparing to pre-existing standardized population norms [29], without adequately controlling for confounding patient demographics and characteristics [8, 28, 29].

The economic burden of NASH reported from previous research shows that NASH is associated with medical resource use and impairment of work and non-work activities [7, 8, 25, 30]. Our findings corroborate the published evidence using a more robust analysis, well defined comparison groups and mirror the results reported in the NHWS EU5 study [10].

As NASH is commonly regarded as an asymptomatic “silent” disease, research that reveals the true burden and impact on patients as well as the possible link between biology, psychological aspects and healthcare utilization is valuable.

This study has some limitations specific to its cross-sectional, real-world design using patient-reported data. All respondents of the NHWS reported their respective condition and their status based on the recall of a physician diagnosis and it was assumed that there was an accurate diagnosis and an accurate recall. This study did not collect data regarding liver biopsy status (i.e., confirmation of NASH via liver biopsy) and some conditions such as obesity were not reported as clinical diagnosis. Respondents reported their BMI which was used as a proxy for obesity. BMI was one of the criteria to obtain the three matched cohorts using propensity score matching. The separate impact that obesity might have on patients’ burden was not an objective of this study. Prior research validates that most patients accurately report their physician-diagnosed chronic conditions [31, 32]. In this study, maybe due to low awareness of NASH and clinical guidelines not recommending routine liver biopsy for all suspected cases, confirmed diagnosis may be more problematic for NASH than for other chronic conditions [33]. Specifically, these results fail to account for individuals who have NASH, but do not report it (either because they are not diagnosed yet or because they do not recall having been diagnosed) or for those who mistakenly report a NASH diagnosis. It seems likely that more respondents fall into the former category (unreported NASH) than the latter category (erroneously reported NASH). However, this could be the case for all the other conditions collected in NHWS. The prevalence of diagnosed NASH observed in these results might be underestimated. On the other hand, one could argue that the burden of NASH observed in these data might have been overestimated. The NASH sample in this study may have been biased toward including patients with more severe NASH since patients with more severe symptoms may be more likely to be identified as patients who warrant consideration for a NASH diagnosis. However, the impact of this potential selection bias on the comparative burden of NASH observed in these data may also be negated by the likely presence of unreported NASH in our non-NASH control cohorts (i.e., T2DM and general population cohorts).

## Conclusion

This study conducted within the NHWS in US as a mirror of the previously published NHWS EU5 [10] indicate that NASH patients in the US encounter a substantial burden of disease which is worse compared to a sample of the general population and at least comparable or worse in some outcomes to T2DM patients. This reveals that NASH might be less a “silent” disease but a disease for which the burden on patients is less recognized. There are currently no approved treatments for NASH aside from diet and exercise, which are often ineffective [34–37]. As disease awareness increases alongside the development of new treatment options, there is an increased need for better characterizing the burden of NASH.

## Supplementary Information

**Additional file 1.**

## Data Availability

The data that support the findings of this study are available from Cerner Enviza (Kantar Health at the time of the analysis) however restrictions apply to the availability of these data, which were used under license for the current study, and so are not publicly available. Data are however available from the corresponding author upon reasonable request and with permission of from Cerner Enviza (Kantar Health at the time of the analysis).

## Abbreviations

BMI: Body mass index
CCI: Charlson Comorbidity Index
EQ VAS: EQ visual analogue scale
EQ-5D-5L: EuroQol 5-Dimension Health Questionnaire
ER: Emergency room
EU5: European Union Five
GP: General practitioner
HRQoL: Health-related quality of life
HRU: Healthcare resource use
M: Adjusted mean
MCS: Mental component summary
MID: Minimal important difference
NAFLD: Nonalcoholic fatty liver disease
NASH: Nonalcoholic steatohepatitis
NHWS: National Health and Wellness Survey
SD: Standard deviation
SE: Standard error
SF-36v2: Revised Medical Outcomes Study 36-Item Short Form Survey Instrument version 2
PCS: Physical component summary
SF-6D: Short Form 6-Dimension
T2DM: type 2 diabetes mellitus
US: United States
WPAI: Work productivity and activity impairment
