# Supplementary material for: The burden of nonalcoholic steatohepatitis (NASH) in the United States
Source: BMC Gastroenterol. 2023 Apr 5;23:109. doi: 10.1186/s12876-023-02726-2 (PMC10077759; doi:10.1186/s12876-023-02726-2)
Supplement: Supplementary file 1 — Additional file 1. [file 12876_2023_2726_MOESM1_ESM.docx]

**SUPPLEMENTARY MATERIALS**

**Demographics**

Demographics assessed included age, gender, race/ethnicity, level of education (university degree/less than university degree), annual household income (below region median, above region median, or decline to answer), and employment status (employed/unemployed).

**Patient Characteristics**

Patient characteristics assessed included health insurance coverage (yes/no), health insurance type (employer sponsored/other/not sure or decline to answer), body mass index (BMI) (underweight [<18.5 kg/m^2^], normal weight [18.5 to <25.0 kg/m^2^], overweight [25.0 to <30.0 kg/m^2^], obese [30.0 kg/m^2^ and above], or unknown), self-reported physician diagnosis of high blood pressure (yes/no), cigarette smoking (current, former, or never), frequency of current alcohol use (none, less than daily, or daily), exercise behavior in the past month: no exercise (0 days), low exercise (1-5 days), moderate exercise (6-11 days), or high exercise (12 days or more), Charlson Comorbidity Index (CCI)^[[1]](#footnote-1)^ and adjusted CCI (see **Methods**), self-reported physician diagnosis of cirrhosis, hepatitis B, or hepatitis C (yes/no), self-reported physician diagnosis of at least one of the following heart or blood conditions: atherosclerosis (coronary artery disease), congestive heart failure, heart attack, stroke, mini-stroke/transient ischemia attack, hypertension (high blood pressure), high cholesterol, peripheral arterial disease, peripheral vascular disease, angina (yes/no), self-reported physician diagnosis of at least one of the following type 2 diabetes mellitus (T2DM)-related complications: macular edema or diabetic retinopathy, kidney disease, foot or leg ulcer, neuropathic pain, end organ damage due to diabetes (yes/no), and current use of the following T2DM prescriptions: insulin prescription, non-insulin prescription, and a prescription (yes/no).

**Healthcare Resource Use Assessed**

Healthcare provider (HCP) visits included the following types of visits: general practitioner/family practitioner, internist, allergist, cardiologist, dentist, dermatologist, diabetologist, endocrinologist, gastroenterologist, geriatrician, gynecologist, hepatologist, infectologist/infectious disease specialist, neurologist, nephrologist, nurse practitioner/physician assistant, obstetrician, oncologist, ophthalmologist, orthopedist, otolaryngologist, plastic surgeon, podiatrist, psychiatrist, psychologist/therapist, pulmonologist, respiratory therapist, rheumatologist, urologist, or other medical specialist.

Non-traditional provider visits included the following types of visits: acupuncturist, chiropractor, herbalist, physical therapist, nutritionist, massage therapist, occupational therapist, pharmacist, pharmacy assistant, homeopath, or other provider.

**Matching Procedure**

Matched T2DM and matched general population cohorts were created via a standard matching procedure. This propensity score matching procedure (1:2 ratio of nonalcoholic steatohepatitis [NASH] patients to T2DM patients; 1:4 ratio of NASH patients to general population), was used to control for any underlying differences in baseline characteristics between these groups. The matching process was based on results from unmatched bivariate comparisons [NASH cohort vs. unmatched T2DM cohort; NASH cohort vs. unmatched general population cohort], whereby relevant and statistically significant (*p*<0.100) variables (i.e., matching criteria) were included in the logistic model used to create propensity scores. Variables that were theoretically important to include as matching criteria (e.g. gender) were also included in the logistic model used to create propensity scores, even if not significant in unmatched bivariate comparisons. A greedy matching algorithm was then applied to the resultant propensity scores to create matched cohorts.

**Supplementary Table 1. HRQoL, WPAI, HRU: NASH vs. matched general population**

|  | | **Matched Cohorts** | | | |  |
| --- | --- | --- | --- | --- | --- | --- |
|  | | **NASH**  **(N=136)** | | **General Population**  **(N=544)** | | ***p*-value** |
|  |  | **Mean (SD)** |  | **Mean (SD)** |  |  |
| **Health-Related Quality of Life (HRQoL)** | | | | | | |
| SF-36v2 | | | | | | |
| *Physical Component Summary (PCS)* | | 42.04 (11.04) | | 47.10 (11.23) | | < 0.001 |
| *Mental Component Summary (MCS)* | | 43.19 (13.00) | | 46.22 (11.99) | | 0.010 |
| *SF-6D health utility score* | | 0.63 (0.13) | | 0.69 (0.14) | | < 0.001 |
| EQ-5D utility score | | 0.72 (0.17) | | 0.78 (0.18) | | < 0.001 |
| Diagnosis of anxiety in past 12 months, N (%) | | 51 (37.5%) | | 139 (25.5%) | | 0.005 |
| Diagnosis of depression in past 12 months, N (%) | | 59 (43.4%) | | 164 (30.1%) | | 0.003 |
| Diagnosis of sleep difficulties (other than insomnia, narcolepsy, or sleep apnea) in past 12 months, N (%) | | 14 (10.3%) | | 48 (8.8%) | | 0.594 |
| **Impairment of Work and Activity** | |  |  |  |  |  |
| WPAI (% score) |  |  |  |  |  |  |
| Absenteeism (employed only) | | 16.96 (28.61) | | 9.19 (21.35) | | 0.012 |
| Presenteeism (employed only) | | 32.92 (30.45) | | 22.20 (28.45) | | 0.007 |
| Overall work impairment (employed only) | | 39.64 (34.80) | | 26.18 (32.93) | | 0.003 |
| Activity impairment | | 44.71 (30.81) | | 30.83 (31.63) | | < 0.001 |
| **Healthcare Resource Use** (past 6 Months) | |  |  |  |  |  |
| HCP visits | | 8.43 (9.63) | | 5.17 (7.32) | | < 0.001 |
| Emergency room visits | | 0.73 (1.91) | | 0.38 (1.23) | | 0.009 |
| Hospitalizations | | 0.43 (1.08) | | 0.21 (0.92) | | 0.013 |

Note: The data captured were **bivariate results**. Tests assume equal variances. NASH, nonalcoholic steatohepatitis; HRQoL, health-related quality of life; WPAI, work productivity and activity impairment; HRU, healthcare resource use; HCP, healthcare professional; SF-36v2, Revised Medical Outcomes Study 36-Item Short Form Survey Instrument; PCS, Physical Component Summary; MCS, Mental Component Summary; M, mean; SD, standard deviation.

**Supplementary Figure 1:** SF-6D and EQ-5D utility scores: NASH vs. matched general population and NASH vs. matched T2DM **
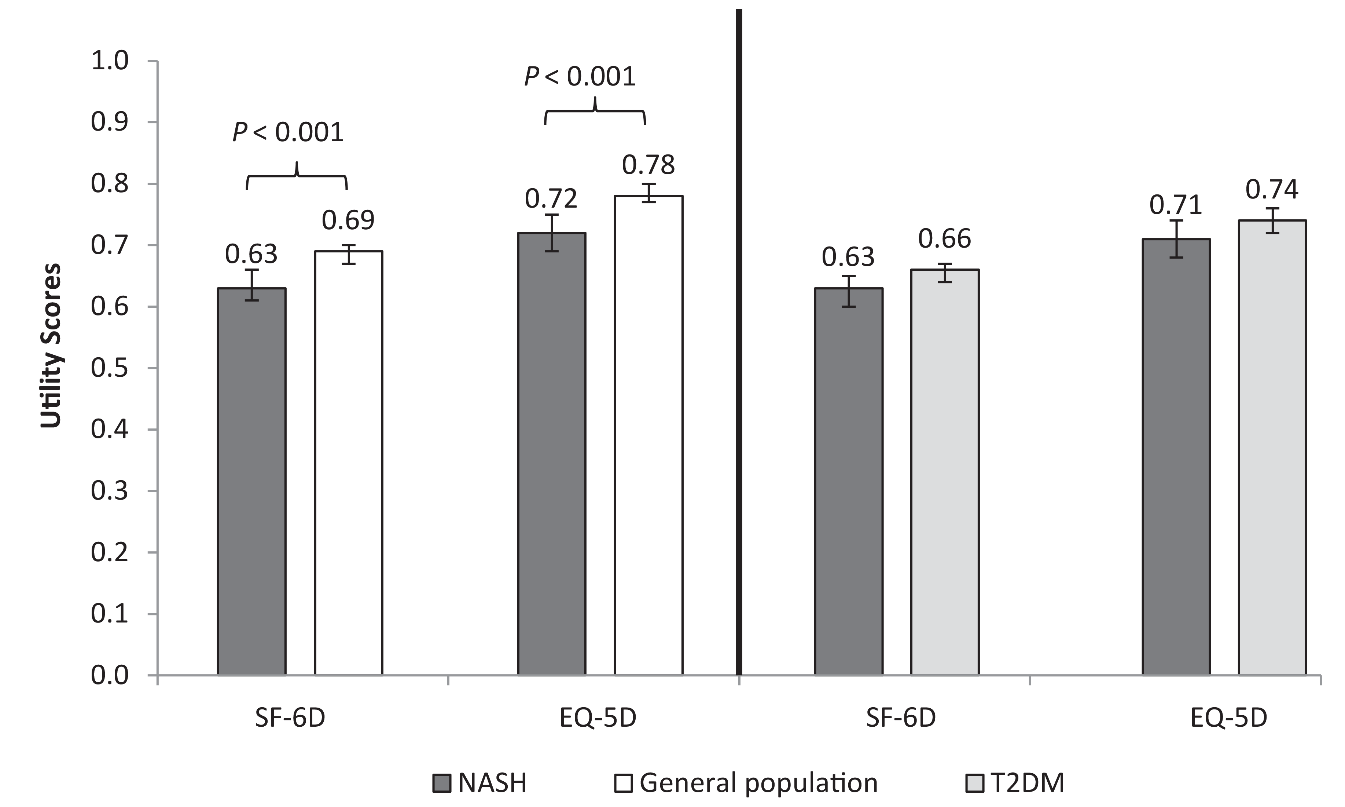
**

Note: The data captured were **multivariable results** displayed as adjusted means. For each comparison, p-values represent significance of the regression coefficient in the regression model with matched general population as reference group and with matched T2DM as reference group, respectively. Abbreviations: EQ-5D, EuroQol 5-Dimension Health Questionnaire; NASH, nonalcoholic steatohepatitis; SF-6D, Short form-6D; T2DM, type 2 diabetes mellitus.

**Supplementary Table 2: SF-6D and EQ-5D utility scores**: (a) NASH vs. matched general population and (b) NASH vs. matched T2DM

| **(a)** | **Matched General Population N=544** | | | | | | **NASH N=136** | | | | | | | | |  |
| --- | --- | --- | --- | --- | --- | --- | --- | --- | --- | --- | --- | --- | --- | --- | --- | --- |
|  | **M** | **SE** | **LCL** | | **UCL** | | **B** | **M** | | **SE** | | **LCL** | | **UCL** | | **p-value** |
| Mental Component Summary (MCS) | 46.22 | 0.52 | 45.19 | | 47.24 | | -3.03 | 43.19 | | 1.04 | | 41.15 | | 45.24 | | .010 |
| Physical Component Summary (PCS) | 47.10 | 0.48 | 46.16 | | 48.04 | | -5.06 | 42.04 | | 0.96 | | 40.16 | | 43.92 | | <.001 |
| SF-6D Utility score | 0.69 | 0.01 | 0.67 | | 0.70 | | -0.05 | 0.63 | | 0.01 | | 0.61 | | 0.66 | | <.001 |
| EQ-5D Utility score | 0.78 | 0.01 | 0.77 | | 0.80 | | -0.06 | 0.72 | | 0.02 | | 0.69 | | 0.75 | | <.001 |
| **(b)** | **Matched T2DM N=272** | | | | | | **NASH N=136** | | | | | | | | |  |
|  | **M** | **SE** | | **LCL** | | **UCL** | **B** | | **M** | | **SE** | | **LCL** | | **UCL** | **p-value** |
| Mental Component Summary (MCS) | 43.89 | 0.81 | | 42.30 | | 45.48 | -0.29 | | 43.60 | | 1.22 | | 41.21 | | 45.99 | .852 |
| Physical Component Summary (PCS) | 44.58 | 0.66 | | 43.29 | | 45.86 | -4.05 | | 40.52 | | 0.98 | | 38.60 | | 42.45 | .001 |
| SF-6D Utility score | 0.66 | 0.01 | | 0.64 | | 0.67 | -0.03 | | 0.63 | | 0.01 | | 0.60 | | 0.65 | .078 |
| EQ-5D Utility score | 0.74 | 0.01 | | 0.72 | | 0.76 | -0.03 | | 0.71 | | 0.02 | | 0.68 | | 0.74 | .098 |
| Note. The data captured were **multivariable results**. No covariates were included in NASH vs. matched general population. In the NASH vs. T2DM covariates included use of insulin prescription to treat T2DM, use of non-insulin prescription to treat T2DM, use of a prescription to treat T2DM, self-reported physician diagnosis of one or more relevant heart or blood conditions, and self-reported physician diagnosis of one or more relevant T2DM-related complications. P-values represent significance of the regression coefficient in the regression model with Matched General Population or matched T2DM as reference group. Abbreviations: M = adjusted mean, SE = standard error, LCL = 95% lower confidence interval, UCL = 95% upper confidence interval, B = unstandardized regression coefficient. | | | | | | | | | | | | | | | | |
| **Supplementary Table 3:** **% report psychological comorbidities**: (a) NASH vs. matched general population (b) NASH vs. matched T2DM   \| **(a)** \| **Matched General Population N=544** \| \| \| \| \| \| **NASH N=136** \| \| \| \| \| \| \| \| \|  \| \| --- \| --- \| --- \| --- \| --- \| --- \| --- \| --- \| --- \| --- \| --- \| --- \| --- \| --- \| --- \| --- \| --- \| \| **Physician diagnosis in past 12 months:** \| **M** \| **SE** \| **LCL** \| \| **UCL** \| \| **OR** \| **M** \| \| **SE** \| \| **LCL** \| \| **UCL** \| \| **p-value** \| \| Anxiety \| 0.26 \| 0.02 \| 0.22 \| \| 0.29 \| \| 1.75 \| 0.38 \| \| 0.04 \| \| 0.30 \| \| 0.46 \| \| .006 \| \| Depression \| 0.30 \| 0.02 \| 0.26 \| \| 0.34 \| \| 1.78 \| 0.43 \| \| 0.04 \| \| 0.35 \| \| 0.52 \| \| .004 \| \| Sleep difficulties, other than insomnia, narcolepsy, or sleep apnea \| 0.09 \| 0.01 \| 0.07 \| \| 0.12 \| \| 1.19 \| 0.10 \| \| 0.03 \| \| 0.06 \| \| 0.17 \| \| .594 \| \| **(b)** \| **Matched T2DM N=272** \| \| \| \| \| \| **NASH N=136** \| \| \| \| \| \| \| \| \|  \| \| **Physician diagnosis in past 12 months:** \| **M** \| **SE** \| \| **LCL** \| \| **UCL** \| **OR** \| \| **M** \| \| **SE** \| \| **LCL** \| \| **UCL** \| **p-value** \| \| Anxiety \| 0.28 \| 0.03 \| \| 0.22 \| \| 0.34 \| 1.73 \| \| 0.40 \| \| 0.05 \| \| 0.31 \| \| 0.50 \| .043 \| \| Depression \| 0.35 \| 0.03 \| \| 0.29 \| \| 0.42 \| 1.53 \| \| 0.45 \| \| 0.05 \| \| 0.36 \| \| 0.55 \| .101 \| \| Sleep difficulties, other than insomnia, narcolepsy, or sleep apnea \| 0.08 \| 0.02 \| \| 0.05 \| \| 0.12 \| 1.27 \| \| 0.09 \| \| 0.03 \| \| 0.05 \| \| 0.17 \| .573 \| \| Note. The data captured were **multivariable results**. No covariates were included in NASH vs. matched general population. In the NASH vs. T2DM covariates included use of insulin prescription to treat T2DM, use of non-insulin prescription to treat T2DM, use of a prescription to treat T2DM, self-reported physician diagnosis of one or more relevant heart or blood conditions, and self-reported physician diagnosis of one or more relevant T2DM-related complications. P-values represent significance of the regression coefficient in the regression model with Matched General Population or matched T2DM as reference group. Abbreviations: M = adjusted mean, SE = standard error, LCL = 95% lower confidence interval, UCL = 95% upper confidence interval, OR = odds ratio. \| \| \| \| \| \| \| \| \| \| \| \| \| \| \| \| \| | | | | | | | | | | | | | | | | |

| **Supplementary Table 4: WPAI scores**: (a) NASH vs. matched general population and (b) NASH vs. matched T2DM   \| **(a)** \| **Matched General Population N=544** \| \| \| \| \| \| **NASH N=136** \| \| \| \| \| \| \| \| \|  \| \| --- \| --- \| --- \| --- \| --- \| --- \| --- \| --- \| --- \| --- \| --- \| --- \| --- \| --- \| --- \| --- \| --- \| \|  \| **M** \| **SE** \| **LCL** \| \| **UCL** \| \| **RR** \| **M** \| \| **SE** \| \| **LCL** \| \| **UCL** \| \| p-value \| \| Absenteeism (employed only) \| 9.19 \| 1.19 \| 7.13 \| \| 11.85 \| \| 1.85 \| 16.97 \| \| 4.61 \| \| 9.97 \| \| 28.89 \| \| .041 \| \| Presenteeism (employed only) \| 22.20 \| 1.60 \| 19.28 \| \| 25.56 \| \| 1.48 \| 32.92 \| \| 5.00 \| \| 24.44 \| \| 44.34 \| \| .019 \| \| Overall work productivity loss (employed only) \| 26.19 \| 1.83 \| 22.84 \| \| 30.02 \| \| 1.51 \| 39.64 \| \| 5.80 \| \| 29.76 \| \| 52.81 \| \| .011 \| \| Activity impairment \| 30.83 \| 1.28 \| 28.42 \| \| 33.44 \| \| 1.45 \| 44.71 \| \| 3.71 \| \| 38.00 \| \| 52.60 \| \| <.001 \| \| **(b)** \| **Matched T2DM N=272** \| \| \| \| \| \| **NASH N=136** \| \| \| \| \| \| \| \| \|  \| \|  \| **M** \| **SE** \| \| **LCL** \| \| **UCL** \| **RR** \| \| **M** \| \| **SE** \| \| **LCL** \| \| **UCL** \| **p-value** \| \| Absenteeism (employed only) \| 8.23 \| 1.83 \| \| 5.33 \| \| 12.73 \| 1.73 \| \| 14.26 \| \| 5.38 \| \| 6.81 \| \| 29.88 \| .283 \| \| Presenteeism (employed only) \| 25.71 \| 2.58 \| \| 21.12 \| \| 31.28 \| 1.29 \| \| 33.18 \| \| 5.35 \| \| 24.18 \| \| 45.52 \| .221 \| \| Overall work productivity loss (employed only) \| 28.61 \| 2.91 \| \| 23.44 \| \| 34.91 \| 1.37 \| \| 39.23 \| \| 6.35 \| \| 28.57 \| \| 53.87 \| .135 \| \| Activity impairment \| 36.07 \| 1.88 \| \| 32.57 \| \| 39.96 \| 1.31 \| \| 47.14 \| \| 3.75 \| \| 40.34 \| \| 55.09 \| .010 \| \| Note. The data captured were **multivariable results**. No covariates were included in NASH vs. matched general population. In the NASH vs. T2DM covariates included use of insulin prescription to treat T2DM, use of non-insulin prescription to treat T2DM, use of a prescription to treat T2DM, self-reported physician diagnosis of one or more relevant heart or blood conditions, and self-reported physician diagnosis of one or more relevant T2DM-related complications. P-values represent significance of the regression coefficient in the regression model with Matched General Population or matched T2DM as reference group. Abbreviations: M = adjusted mean, SE = standard error, LCL = 95% lower confidence interval, UCL = 95% upper confidence interval, RR = rate ratio. \| \| \| \| \| \| \| \| \| \| \| \| \| \| \| \| \|   **Supplementary Table 5. HCP specialty visits**: NASH vs. matched general population   \|  \| **Matched Cohorts** \| \|  \| \| --- \| --- \| --- \| --- \| \|  \| **NASH**  **N=136** \| **Matched General Population**  **N=544** \|  \| \|  \| **M (SE)** \| **M (SE)** \| ***p*-value** \| \| **General / Family Medicine**  **provider visits** \| **1.49 (0.17)** \| **1.14 (0.07)** \| **0.040** \| \| **Specialists (any type) visits** \| **6.95 (0.96)** \| **4.03 (0.29)** \| **< 0.001** \| \| Cardiologist visits \| 0.26 (0.07) \| 0.19 (0.03) \| 0.282 \| \| Gastroenterologist visits \| 0.38 (0.08) \| 0.12 (0.02) \| < 0.001 \| \| Endocrinologist visits \| 0.21 (0.07) \| 0.11 (0.02) \| 0.068 \| \| Internist visits \| 0.40 (0.10) \| 0.21 (0.03) \| 0.020 \| \| Diabetologist visits \| 0.01 (0.01) \| 0.04 (0.01) \| 0.299 \| \| Psychiatrist visits \| 0.32 (0.10) \| 0.27 (0.05) \| 0.687 \| \| Hepatologist visits \| 0.10 (0.05) \| 0.02 (0.01) \| 0.022 \|   Note: The data captured were **multivariable results**. The data above is self-reported healthcare resource use in the past six months. P-values represent significance of the regression coefficient in the regression model with matched general population as reference group. Abbreviations: HRU, healthcare resource use; NASH, nonalcoholic steatohepatitis. M, adjusted mean; SE, standard error.  **Supplementary Table 6: Healthcare resource use**: (a) NASH vs. matched general population; (b) NASH vs. matched T2DM   \| **(a)** \| **Matched General Population N=544** \| \| \| \| \| \| **NASH N=136** \| \| \| \| \| \| \| \| \|  \| \| --- \| --- \| --- \| --- \| --- \| --- \| --- \| --- \| --- \| --- \| --- \| --- \| --- \| --- \| --- \| --- \| --- \| \|  \| **M** \| **SE** \| **LCL** \| \| **UCL** \| \| **RR** \| **M** \| \| **SE** \| \| **LCL** \| \| **UCL** \| \| **p-value** \| \| Number of traditional HCP visits \| 5.17 \| 0.30 \| 4.61 \| \| 5.80 \| \| 1.63 \| 8.43 \| \| 0.96 \| \| 6.75 \| \| 10.54 \| \| <.001 \| \| Number of ER visits \| 0.38 \| 0.05 \| 0.29 \| \| 0.50 \| \| 1.91 \| 0.73 \| \| 0.18 \| \| 0.45 \| \| 1.18 \| \| .021 \| \| Number of hospitalizations \| 0.21 \| 0.04 \| 0.14 \| \| 0.29 \| \| 2.11 \| 0.43 \| \| 0.12 \| \| 0.25 \| \| 0.74 \| \| .024 \| \| Number of different types of non-traditional providers visited \| 0.66 \| 0.04 \| 0.59 \| \| 0.74 \| \| 1.83 \| 1.21 \| \| 0.11 \| \| 1.01 \| \| 1.44 \| \| <.001 \| \| **(b)** \| **Matched T2DM N=272** \| \| \| \| \| \| **NASH N=136** \| \| \| \| \| \| \| \| \|  \| \|  \| **M** \| **SE** \| \| **LCL** \| \| **UCL** \| **RR** \| \| **M** \| \| **SE** \| \| **LCL** \| \| **UCL** \| **p-value** \| \| Number of traditional HCP visits \| 5.68 \| 0.41 \| \| 4.93 \| \| 6.54 \| 1.52 \| \| 8.63 \| \| 0.92 \| \| 7.00 \| \| 10.63 \| .003 \| \| Number of ER visits \| 0.41 \| 0.08 \| \| 0.28 \| \| 0.60 \| 0.99 \| \| 0.41 \| \| 0.12 \| \| 0.24 \| \| 0.71 \| .986 \| \| Number of hospitalizations \| 0.16 \| 0.04 \| \| 0.09 \| \| 0.26 \| 1.22 \| \| 0.19 \| \| 0.07 \| \| 0.10 \| \| 0.38 \| .625 \| \| Number of different types of non-traditional providers visited \| 0.72 \| 0.06 \| \| 0.61 \| \| 0.85 \| 1.54 \| \| 1.11 \| \| 0.12 \| \| 0.89 \| \| 1.38 \| .005 \| \| Note. The data captured were **multivariable results** reported in the past 6 months. No covariates were included in NASH vs. matched general population. In the NASH vs. T2DM covariates included use of insulin prescription to treat T2DM, use of non-insulin prescription to treat T2DM, use of a prescription to treat T2DM, self-reported physician diagnosis of one or more relevant heart or blood conditions, and self-reported physician diagnosis of one or more relevant T2DM-related complications. P-values represent significance of the regression coefficient in the regression model with Matched General Population or matched T2DM as reference group. Abbreviations: M = adjusted mean, SE = standard error, LCL = 95% lower confidence interval, UCL = 95% upper confidence interval, RR = rate ratio. \| \| \| \| \| \| \| \| \| \| \| \| \| \| \| \| \| |
| --- | --- | --- | --- | --- | --- | --- | --- | --- | --- | --- | --- | --- | --- | --- | --- | --- | --- | --- | --- | --- | --- | --- | --- | --- | --- | --- | --- | --- | --- | --- | --- | --- | --- | --- | --- | --- | --- | --- | --- | --- | --- | --- | --- | --- | --- | --- | --- | --- | --- | --- | --- | --- | --- | --- | --- | --- | --- | --- | --- | --- | --- | --- | --- | --- | --- | --- | --- | --- | --- | --- | --- | --- | --- | --- | --- | --- | --- | --- | --- | --- | --- | --- | --- | --- | --- | --- | --- | --- | --- | --- | --- | --- | --- | --- | --- | --- | --- | --- | --- | --- | --- | --- | --- | --- | --- | --- | --- | --- | --- | --- | --- | --- | --- | --- | --- | --- | --- | --- | --- | --- | --- | --- | --- | --- | --- | --- | --- | --- | --- | --- | --- | --- | --- | --- | --- | --- | --- | --- | --- | --- | --- | --- | --- | --- | --- | --- | --- | --- | --- | --- | --- | --- | --- | --- | --- | --- | --- | --- | --- | --- | --- | --- | --- | --- | --- | --- | --- | --- | --- | --- | --- | --- | --- | --- | --- | --- | --- | --- | --- | --- | --- | --- | --- | --- | --- | --- | --- | --- | --- | --- | --- | --- | --- | --- | --- | --- | --- | --- | --- | --- | --- | --- | --- | --- | --- | --- | --- | --- | --- | --- | --- | --- | --- | --- | --- | --- | --- | --- | --- | --- | --- | --- | --- | --- | --- | --- | --- | --- | --- | --- | --- | --- | --- | --- | --- | --- | --- | --- | --- | --- | --- | --- | --- | --- | --- | --- | --- | --- | --- | --- | --- | --- | --- | --- | --- | --- | --- | --- | --- | --- | --- | --- | --- | --- | --- | --- | --- | --- | --- | --- | --- | --- | --- | --- | --- | --- | --- | --- | --- | --- | --- | --- | --- | --- | --- | --- | --- | --- | --- | --- | --- | --- | --- | --- | --- | --- | --- | --- | --- | --- | --- | --- | --- | --- | --- | --- | --- | --- | --- | --- | --- | --- | --- | --- | --- | --- | --- | --- | --- | --- | --- | --- | --- | --- | --- | --- | --- | --- | --- | --- | --- | --- | --- | --- | --- | --- | --- | --- | --- | --- | --- | --- | --- | --- | --- | --- | --- | --- | --- | --- | --- | --- | --- | --- | --- | --- | --- | --- | --- | --- | --- | --- | --- | --- | --- | --- | --- | --- | --- | --- | --- | --- | --- | --- | --- | --- | --- | --- | --- | --- | --- | --- | --- | --- | --- | --- | --- | --- | --- | --- | --- | --- | --- | --- | --- | --- | --- | --- | --- | --- | --- | --- | --- | --- | --- | --- | --- | --- | --- | --- | --- | --- | --- | --- | --- | --- | --- | --- | --- | --- | --- | --- | --- | --- | --- | --- | --- | --- | --- | --- | --- | --- | --- | --- | --- | --- | --- | --- | --- | --- | --- | --- | --- | --- | --- | --- | --- | --- | --- | --- | --- | --- | --- | --- | --- | --- | --- | --- | --- | --- | --- | --- | --- | --- | --- | --- | --- | --- | --- | --- | --- | --- | --- | --- | --- | --- | --- | --- | --- | --- | --- | --- | --- | --- | --- | --- | --- | --- | --- | --- |

**Supplementary Table 7. HRQoL, WPAI, and HRU: NASH vs. matched T2DM**

|  | **Matched Cohorts** | | | |  |
| --- | --- | --- | --- | --- | --- |
|  | **NASH**  **(N=136)** | | **T2DM**  **(N=272)** | | ***p*-value** |
|  | **Mean (SD)** |  | **Mean (SD)** |  |  |
| **Health-Related Quality of Life (HRQoL)** | | | | | |
| SF-36v2 | | | | | |
| *Physical Component Summary (PCS)* | 42.04 (11.04) | | 43.82 (11.06) | | 0.126 |
| *Mental Component Summary (MCS)* | 43.19 (13.00) | | 44.10 (12.53) | | 0.498 |
| *SF-6D health utility score* | 0.63 (0.13) | | 0.66 (0.14) | | 0.091 |
| EQ-5D utility score | 0.72 (0.17) | | 0.74 (0.17) | | 0.234 |
| Diagnosis of anxiety in past 12 months,  N (%) | 51 (37.5%) | | 79 (29.0%) | | 0.084 |
| Diagnosis depression in past 12 months,  N (%) | 59 (43.4%) | | 99 (36.4%) | | 0.172 |
| Diagnosis of sleep difficulties (other than insomnia, narcolepsy, or sleep apnea) in past 12 months, N (%) | 14 (10.3%) | | 21 (7.7%) | | 0.382 |
| **Impairment of Work and Activity** | | | | | |
| WPAI (% impairment score) | | | | | |
| Absenteeism (employed only) | 16.96 (28.61) | | 9.16 (18.30) | | 0.019 |
| Presenteeism (employed only) | 32.92 (30.45) | | 27.73 (29.07) | | 0.242 |
| Overall work impairment (employed only) | 39.64 (34.80) | | 30.96 (32.70) | | 0.081 |
| Activity impairment | 44.71 (30.81) | | 38.64 (30.95) | | 0.062 |
| **Healthcare Resource Use in Past Six Months** |  |  |  |  |  |
| HCP visits | 8.43 (9.63) | | 5.90 (6.32) | | 0.002 |
| Emergency room visits | 0.73 (1.91) | | 0.48 (1.92) | | 0.215 |
| Hospitalizations | 0.43 (1.08) | | 0.29 (1.27) | | 0.260 |

Note: The data captured were **bivariate results.** Tests assume equal variances. NASH, nonalcoholic steatohepatitis; T2DM, type 2 diabetes mellitus; HRQoL, health-related quality of life; WPAI, work productivity and activity impairment; HRU, healthcare resource use; HCP, healthcare professional; SF-36v2, Revised Medical Outcomes Study 36-Item Short Form Survey Instrument; PCS, Physical Component Summary; MCS, Mental Component Summary; M, mean; SD, standard deviation.

**Supplementary Table 8. HCP specialty visits**: NASH vs. matched T2DM

|  | **Matched Cohorts** | |  |
| --- | --- | --- | --- |
|  | **NASH**  **N=136** | **Matched T2DM**  **N=272** |  |
|  | **M (SE)** | **M (SE)** | ***p*-value** |
| **General / Family Medicine provider visits** | **1.60 (0.20)** | **1.62 (0.13)** | **0.955** |
| **Specialists’ visits (any)** | **6.97 (0.92)** | **3.98 (0.36)** | **0.001** |
| *Cardiologist visits* | 0.30 (0.08) | 0.16 (0.03) | 0.098 |
| *Gastroenterologist visits* | 0.31 (0.09) | 0.15 (0.04) | 0.069 |
| *Endocrinologist visits* | 0.23 (0.06) | 0.21 (0.04) | 0.808 |
| *Internist visits* | 0.34 (0.10) | 0.30 (0.06) | 0.766 |
| *Diabetologist visits* | 0.01 (0.01) | 0.07 (0.03) | 0.143 |
| *Psychiatrist visits* | 0.34 (0.14) | 0.21 (0.07) | 0.408 |
| *Hepatologist visits* | 0.01 (0.01) | 0.00 (0.00) | 0.053 |

Note: The data captured were **multivariable results**. The data above is self-reported healthcare resource use in the past six months. P-values represent significance of the regression coefficient in the regression model with matched T2DM as reference group. Abbreviations: HRU, healthcare resource use; NASH, nonalcoholic steatohepatitis; T2DM, type 2 diabetes mellitus; M, adjusted mean; SE, standard error.

1. Charlson ME, Pompei P, Ales KL, MacKenzie CR. A new method of classifying prognostic comorbidity in longitudinal studies: development and validation. J Chronic Dis 1987;40(5):373-383. doi:10.1016/0021-9681(87)90171-8  [↑](#footnote-ref-1)
